# Downregulated Long Noncoding RNA BANCR Promotes the Proliferation of Colorectal Cancer Cells via Downregualtion of p21 Expression

**DOI:** 10.1371/journal.pone.0122679

**Published:** 2015-04-30

**Authors:** Yongguo Shi, Yangchen Liu, Jirong Wang, Ding Jie, Tian Yun, Wang Li, Lin Yan, Keming Wang, Jifeng Feng

**Affiliations:** 1 Department of Oncology, Second Affiliated Hospital, Nanjing Medical University, Nanjing, Jiangsu, PR China; 2 Taixing People's Hospital, Taixing, Jiangsu, PR China; 3 Cancer Hospital of Jiangsu Province, Nanjing, Jiangsu, PR China; CSIR Institute of Genomics and Integrative Biology, INDIA

## Abstract

BRAF activated non-coding RNA (BANCR), a long non-coding RNA (lncRNA), is crucial for cell migration in melanoma cells and non-small cell lung cancer (NSCLC) cells. However, little is known regarding the role of this gene in the proliferation of colorectal cancer. Therefore, we investigated the involvement of BANCR in the proliferation of colorectal cancer cells. In this study, we show that BANCR expression was significantly down-regulated in colorectal cancer tissues compared with normal tissues, and overexpression of BANCR suppressed colorectal cancer cell growth *in vitro* and *in vivo*. We also determined that pCDNA-BANCR-mediated colorectal cancer cell proliferation was associated with induction of G0/G1 cell-cycle arrest and apoptosis enhancement through regulation of p21, and its effects were most likely posttranscriptional. Taken together, our findings suggest that down-regulation of BANCR contributes to the proliferation of colorectal cancer cells, at least in part, through the regulation of p21 protein.

## Introduction

Colorectal cancer (CRC) is the third most common type of cancer worldwide [1]. Despite surgical resection and advances in radiotherapy and chemotherapy, it remains the third leading cause of cancer death in men and women in the United States [2]. Because numerous colorectal cancer patients with advanced disease fail to respond well to current treatment regimens, further research regarding the oncogenic signaling mechanisms underlying colorectal cancer that refine the existing programs and new therapeutic targets for the treatment of this disease are urgently needed.

The lncRNAs are important new members of the family of ncRNAs, which are greater than 200 nt and are unable to be translated into proteins[3]. Emerging evidence suggests that lncRNAs may serve as master gene regulators capable of controlling protein-coding and non-coding genes[4]. Dysfunction of these genes has been strongly associated with cell fate determination and human disease pathogenesis, including cancer[5]. For example, HOTAIR is highly expressed in breast tumors[6], MEG3 is significantly down-regulated in NSCLC tissues[7], and aberrant expression of LOC285194 is observed in colorectal cancer [8]. These findings suggest that lncRNAs may serve as important regulators in tumorigenesis, and their molecular and biological functions are important for understanding the molecular biology of cancer progression, including colorectal cancer.

BANCR, an lncRNA originally identified in melanoma cells[9] 693 bp in length, is highly expressed in melanoma cells and is crucial for melanoma cell migration[9]. Subsequently, Sun et al. investigated the effects of BANCR expression on NSCLC cell phenotypes in vitro and in vivo, and they demonstrated that alteration of BANCR expression influenced E-cadherin, N-cadherin and vimentin protein levels[10]. In this study, we assessed the effects of BANCR expression on colorectal cancer cell phenotypes in vitro and in vivo, and we showed that BANCR suppressed colorectal cancer cell growth through interaction with P21 protein. This study advances our understanding of the role of BANCR as a regulator of the pathogenesis of colorectal cancer and facilitates the development of lncRNA for future studies in colorectal cancer.

## Materials and Methods

### Tissue collection

A total of 38 fresh colorectal cancer tissue samples and paired adjacent noncancerous tissue samples were obtained from patients who had undergone surgical resection of colorectal cancer between 2010 and 2012 at the Second Affiliated Hospital of Nanjing Medical, China. The colorectal cancer diagnosis was histopathologically confirmed, and neither local nor systemic treatment had been administered to these patients prior to the operation. The pathological stage, grade and nodal status were appraised by an experienced pathologist. Clinicopathologic characteristics including tumor-node-metastasis (TNM) staging were also collected. The non-tumorous tissues were 5 cm from the edge of the tumor and there were no obvious tumor cells, as evaluated by a pathologist. The clinical information for all of the samples is detailed in Table 1. All of the tissue samples were washed with sterile phosphate-buffered saline before being snap frozen in liquid nitrogen and stored at -80°C until total RNA was extracted. All patients or their guardians provided written informed consent. An approval was obtained from the Research Ethics Committee of Nanjing Medical University, China.

**Table 1:** The Clinical Characteristics of the CRC Patients.

Variables	Number (n = 38)	Percent
**Age(years)**		
<60	16	42.1
≥60	22	57.9
**Gender**		
Male	20	52.6
Female	18	47.4
**Maximum tumor size**		
<5cm	28	73.7
≥5cm	10	26.3
**Location**		
Colon	13	34.2
Rectum	25	65.8
**Depth of tumor**		
T1 and T2	15	39.5
T3 and T4	23	60.5
**Tumor stage**		
I and II	27	71.1
III and IV	11	28.9
**Lymph node metastasis**		
Negative	21	55.3
Positive	17	44.7

### Ethics statement

The study was approved by the Ethics Committee of Nanjing Medical University and it was performed in compliance with the Helsinki Declaration. Written informed consent was obtained for all patient samples. All experimental animals were housed under specific pathogen-free conditions. All experimental procedures were approved by the Institutional Review Board of the Nanjing Medical University. All procedures were performed in accordance with the Nanjing Medical University Guide for the Care and Use of Laboratory Animals formulated by the National Society for Medical Research.

### Cell line and culture conditions

The human colorectal cancer cell lines SW480, HCT116, RQO and HT-29 and the human colonic epithelial cells HCoEpiC were obtained from American Type Culture Collection (Manassas, VA). All of the cell lines were grown and maintained in RPMI Medium 1640 (Invitrogen) supplemented with 10% fetal bovine serum, 100U/ml penicillin, and 100 mg/ml streptomycin (Invitrogen, Shanghai, China) at 37°C in a 5% CO_2_ atmosphere.

### RNA extraction and qRT-PCR analyses

Total RNA was extracted from tissue samples and cultured cells with TRIzol reagent (Invitrogen Life Technologies). RNA from all cells and tissues was reverse transcribed to cDNA from 500 ng of total RNA in a final volume of 10μl using a standard protocol from Power SYBR Green (Takara). For qRT-PCR, cDNA (500 ng) was amplified using BANCR forward primer (5’- ACAGGACTCCATGGCAAACG -3’) and BANCR reverse primer (5’- ATGAAGAAAGCCTGGTGCAGT -3’). qRT-PCR analyses were performed using a standard protocol from Power SYBR Green (Takara). All protocols were performed according to the manufacturer’s instructions. Standard curves were set up for all the tissue samples and cultured cells by making a 100-fold dilution series, starting from 5 ng to 5 fg cDNA. ΔCt values were normalized to glyceraldehyde-3-phosphate dehydrogenase (GAPDH). The primer sequences was as follows: GAPDH: Forward: 5’-AGAGGCAGGGATGATGTTCTG-3’; Reverse:5’-GACTCATGACCACAGTCCATGC-3’. Each sample was analyzed in triplicate.

### Plasmid DNA Transfection

The BANCR sequence was synthesized and subcloned into pCDNA3.1 (Invitrogen, Shanghai, China) vector. The pCDNA-BANCR or empty vector was transfected into SW480 and HCT116 cells cultured in six-well plates according to the manufacturer’s instructions. The empty vector was used as the control. The expression level of BANCR was detected by qRT-PCR.

### RNA Interference by shRNA

Stable BANCR or p21 knockdown SW480 and HCT116 cells were generated by transfection of shRNA. Empty pCDNA was used as the control. The BANCR-shRNAs or P21-shRNA were transfected into SW480 and HCT116 cells cultured in six-well plates using Lipofectamine 2000 (Invitrogen, Shanghai, China), according to the manufacturer’s instructions. The cells were harvested 48 h after transfection, and the total RNA was isolated using TRIZOL reagent (Invitrogen, Carlsbad, CA) according to the manufacturer’s instructions. The specific silencing of BANCR and P21 expression was assessed using qRT-PCR.

### Determination of Cell Viability and Colony Formation Assay

Cell proliferation was monitored using the Cell Proliferation Reagent Kit I (MTT; Roche Applied Science). 3000 cells per well were allowed to grow in 96-well plates with five replicate wells. Cell proliferation was measured every 24 hours following the manufacturer’s protocol. For the colony formation assay, a total of 500 cells were plated into each well of a 6-well plate and maintained in media containing 10% FBS to allow colony formation for 2 weeks incubation at 37.0°C in 5% CO2, replacing the medium every 4 days. The colonies were fixed with methanol, stained with Giemsa, and counted. Triplicate wells were measured in each treatment group.

### Flow-Cytometric Analysis of Cell Cycle and Apoptosis

The pCDNA-BANCR- or empty vector-transfected SW480 and HCT116 cells were cultured in six-well plates for 48 h, washed with ice-cold phosphate-buffered saline, and fixed with 75% ethanol overnight. The cells were then collected by centrifugation (2500 rpm for 3 min) and resuspended in 1 mL of PI solution (50 mg/mL in PBS) containing 0.25 mg/mL of RNase A. After incubation for 15 min in the dark at 4°C, the cells were analyzed by flow cytometry (FACScan; BD Biosciences) using an instrument equipped with Cell Quest software (BD Biosciences). The percentages of the cells in G0–G1, S, and G2–M phases were counted and compared.

The pCDNA-BANCR- or empty vector-transfected SW480 and HCT116 cells were harvested 48 h after transfection for apoptosis analysis. Floating and adherent cells were collected using 0.1% trypsin, washed twice with cold PBS, and suspended in 1000 mL binding buffer (10 mmol/L HEPES buffer, pH 7.4, 140 mmol/L NaCl, and 2.5 mmol/L CaCl_2_). The cells were then treated with fluorescein isothio-cyanate (FITC)-Annexin V and propidium iodide (PI) in the dark at room temperature, according to the manufacturer’s recommendations. The cells were then examined by flow cytometry (FACScan; BD Biosciences) on in instrument equipped with CellQuest software (BD Biosciences), and they were discriminated into viable cells, dead cells, early apoptotic cells, and late apoptotic cells. The percentage of early and late apoptotic cells was compared with control groups from each experiment. This assay was repeated three times.

### Western Blot Assay and Antibodies

Western blot analysis was performed as previously described[7]. Briefly, cellular protein lysates were separated by 10% sodium dodecyl sulfate polyacrylamide gel electrophoresis (SDS-PAGE), then transferred to 0.22μm NC membranes (Sigma) and incubated with specific antibodies. ECL chromogenic substrate was used and signals were quantified by densitometry (Quantity One software, Bio-Rad). Mean ± SD was calculated from 3 individual experiments. GAPDH antibody was used as a control; anti-P21 (1:1,000) and was purchased from Cell Signaling Technology, Inc. (CST), anti-GAPDH (1:1,000) antibody was purchased from Sigma-Aldrich (USA).

### Mouse Xenograft Studies

Four-week-old male BALB/c nude mice were obtained from Shanghai Laboratory Animals Center of the Chinese Academy of Sciences (Shanghai, China). HCT116 cells were transfected with pCDNA-BANCR or empty vector and harvested from six-well cell culture plates. Then, the cells were washed with PBS and resuspended at a concentration of 5×10^7^ cells/mL. Each mouse was injected into the lower right flank with 5×10^6^ suspended cells. Tumor growth was monitored by calipers every three days, and tumor volumes were calculated according to the following formula: 0.5 × length × width^2^. Mice were sacrificed at 2 weeks post-injection by CO2 asphyxiation. The tumors were removed from all of the animals for further analysis.

### Histopathological and immunohistochemical analysis

The fixed tissue specimens embedded in paraffin were cut into 4-mm-thick slices for hematoxylin and eosin (H&E) staining and immunohistochemical (IHC) analysis[11]. Following deparaffinization, dehydration, and antigen retrieval, one section for each sample was stained with hematoxylin and eosin (H&E). For immunohistochemical studies, the specimens were incubated with anti-Ki67 and P21 antibodies against human targets at 4°C overnight. After that, the samples were washed with PBS and incubated at 37°C in a water bath for 2 h after dropwise addition of the secondary antibody and washing with PBS. After treatment with the DAB solution, the samples were flushed completely, counterstained with hematoxylin, washed with water, treated with dehydration and transparency media, mounted on slides, and observed under the microscope. The IHC staining results were scored by the pathologist and author separately to minimize the subjective factors, compared, and the final comprehensive results were obtained.

### Ethynyl deoxyuridine (Edu) Analysis

Proliferating cells were determined by using the 5-ethynyl-2’-deoxyuridine (EdU) labeling/detection kit (Ribobio, Guangzhou, China) according to the manufacturer’s protocol. Briefly, SW480 and HCT116 cells were cultured in 96-well plates at 5 × 10^3^ cells per well, transfected with pCDNA-BANCR or empty vector for 48 h, then 50μM EdU labeling medium was added to the cell culture and allowed to incubate for 2 h at 37°C under 5% CO2. Afterwards, cultured cells were fixed with 4% paraformaldehyde (pH 7.4) for 30 min and treated with 0.5% Triton X-100 for 20 min at room temperature. After washing with PBS, staining with anti-EdU working solution was performed at room temperature for 30 min. Subsequently, the cells were incubated with 100 μL Hoechst 33342 (5 μg/mL) at room temperature for 30 min, followed by observation under a fluorescent microscope. The percentage of EdU-positive cells was calculated from five random fields in three wells.

### Statistical Analysis

Statistical analysis was performed using SPSS software (SPSS, Inc., Chicago, IL, USA). For comparisons between two samples, an unpaired two-tailed *t*-test was performed. A *p*-value of < 0.05 was considered statistically significant. The results are reported as the mean ± SD. Statistical significance was assigned at P < 0.05 (*) or P < 0.01 (**). All experiments were performed at least three times.

## Results

### Expression of BANCR in colorectal cancer tissues and colorectal cancer cell lines

**Fig 1:**
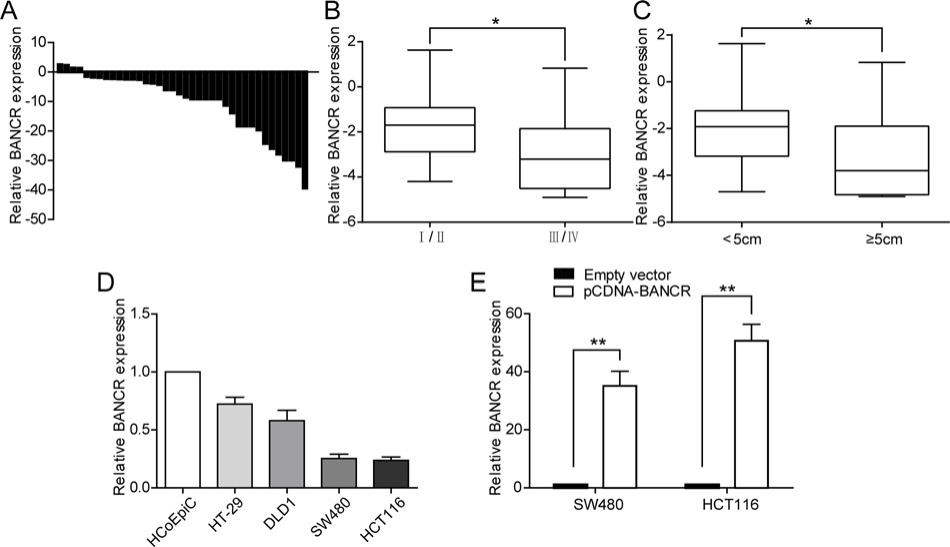
Relative expression of BANCR in colorectal cancer tissues and colorectal cancer cell lines. (A) Relative expression of BANCR in colorectal cancer tissues (n = 38) compared with corresponding non-tumor tissues (n = 38). BANCR expression was examined by qPCR and normalized to GAPDH expression. Results were presented as the fold-change in tumor tissues relative to normal tissues. (B and C) Data are presented as relative expression level in tumor tissues. BANCR expression was significantly lower in patients with a higher pathological stage and larger tumor size (shown as ΔCT). (D) BANCR expression levels of colorectal cancer cell lines (HT-29, DLD1, SW480, and HCT116) compared with the human colonic epithelial cells (HCoEpiC). (E) qPCR analysis of BANCR expression levels following the treatment of SW480 and HCT116 cells with pCDNA-BANCR and empty vector.*, P < 0.05 and **, P < 0.01.

qRT-PCR analysis was used to examine BANCR levels in 38 colorectal cancer tissues and 38 matched normal colorectal tissues. Significantly, the expression of BANCR was down-regulated in colorectal cancer tissues compared with normal tissues (Fig 1A). Furthermore, we evaluated the correlation of BANCR expression with clinicopathological parameters (i.e., stage, maximum diameter) to assess its clinical significance. As presented in Fig 1B and 1C, larger tumors, which represent a higher tumor burden, or more advanced tumors had lower BANCR expression. These analyses demonstrate that BANCR may be a good diagnostic biomarker for early-stage colorectal cancer. Next, BANCR expression was detected by qRT-PCR in 4 human colorectal cancer cell lines (Fig 1D). Notably, all the cell lines expressed lower levels of BANCR versus the human colonic epithelial cells (HCoEpiC), but SW480 and HCT116 cells expressed relatively lower levels of BANCR compared with HT-29 and DLD1 cells. Therefore, we chose SW480 and HCT116 for further studies. To assess the transfection efficiency, BANCR was overexpressed in SW480 and HCT116 cells by transfecting them with pCDNA-BANCR. qRT-PCR analysis of BANCR levels revealed that BANCR expression was increased by 35-fold and 50-fold in SW480 and HCT116 cells, respectively, following transfection with pCDNA-BANCR compared with control (Fig 1E).

### The Effect of BANCR on Cell Proliferation in Colorectal Cancer Cell Lines

**Fig 2:**
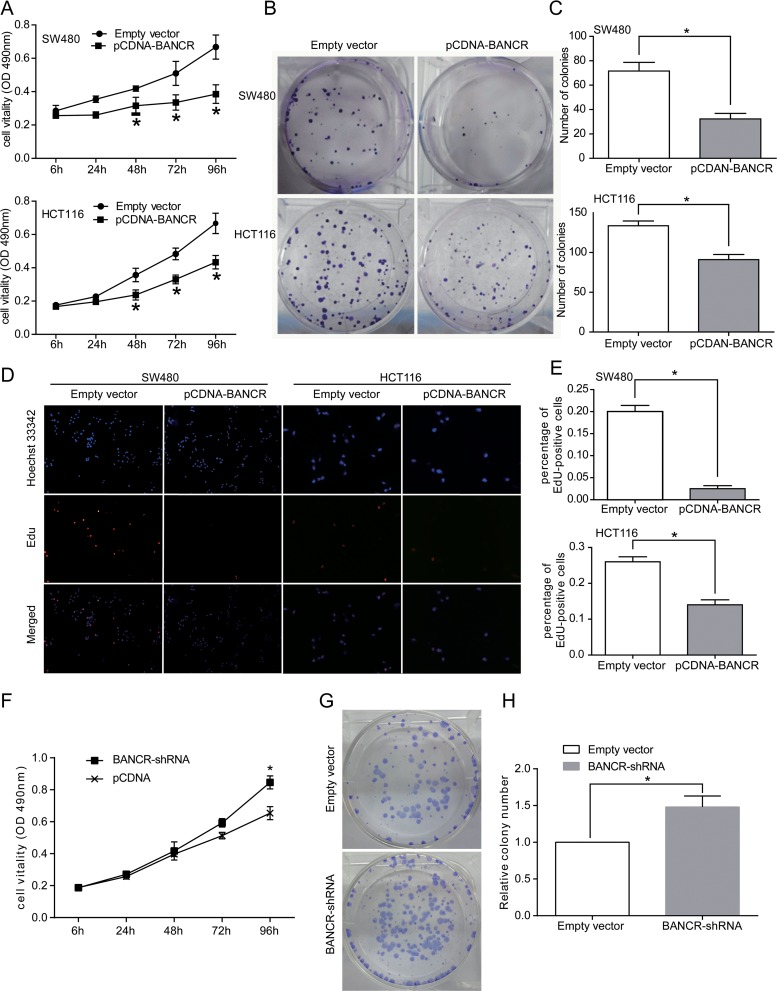
Effects of BANCR on colorectal cancer cell proliferation in vitro. (A) MTT assay was performed to determine the proliferation of SW480 and HCT116 cells. Data represent the mean ± S.D. from three independent experiments. (B and C) Colony-forming growth assays were performed to determine the proliferation of SW480 and HCT116 cells. The colonies were counted and captured. (D and E) Proliferating SW480 and HCT116 cells were labeled with EdU. The Click-it reaction revealed EdU staining (red). Cell nuclei were stained with Hoechst 33342 (blue). The images are representative of the results obtained. (F) MTT assay was performed to determine the proliferation of HCT116 cells. Data represent the mean ± S.D. from three independent experiments. (G and H) Colony-forming growth assays were performed to determine the proliferation of HCT116 cells. The colonies were counted and captured. *,P < 0.05.

To investigate the biological role of BANCR in colorectal cancer, MTT assays were used to detect the impact of BANCR over-expression on proliferation of SW480 and HCT116 colorectal cancer cells. According to the results, we found that the growth of SW480 and HCT116 cells transfected with pCDNA-BANCR was impaired compared with that of control cells (p < 0.05; Fig 2A). To further examine the proliferative effect of BANCR on the growth of SW480 and HCT116 cells, colony formation assays were performed. The results revealed that the colony numbers of colorectal cancer cells transfected with pCDNA-BANCR were obvious lower than those negative control (P < 0.05; Fig 2B and 2C). EdU (red)/Hoechst (blue) immunostaining further confirmed this comparison (Fig 2D and 2E). These findings suggest that BANCR is involved in colorectal cancer cell proliferation.

### BANCR Knockdown Mildly Promoted Proliferation in HCT116 Cells

Our clinical data indicated that BANCR expression was inversely related to colorectal cancer progression. Thus, we used shRNAs to down-regulate endogenous BANCR expression in the HCT116 cell line. An MTT assay was performed as described previously to monitor the effect of BANCR shRNA on cell proliferation at different times, and a colony-forming assay was also performed. The cell proliferation rate when treated with the BANCR shRNA was higher than observed in controls infected with the empty vector at 96 h (Fig 2F). Additionally, the BANCR shRNA also mildly protected the colony-forming ability compared with negative controls (Fig 2G and 2H). The observations made with the shRNA indicated that lower BANCR expression may contribute to colorectal cancer progression.

### BANCR promotes G1 arrest and causes apoptosis in colorectal cancer cells

**Fig 3:**
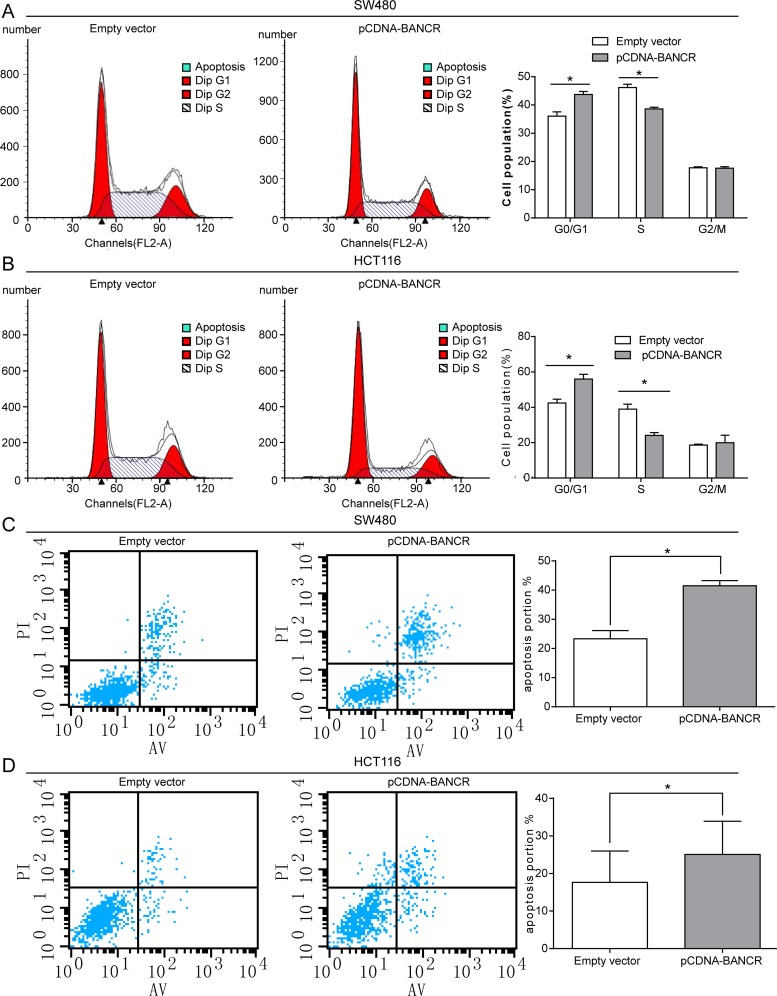
The effect of BANCR on colorectal cancer cell cycle and apoptosis in vitro. (A, B) The bar chart represents the percentage of cells in G0/G1, S, or G2/M phase, as indicated. (C, D) The percentage of apoptotic cells was determined by flow-cytometric analysis. The data represent the mean ± SD from three independent experiments. *, P < 0.05.

To determine whether the effects of BANCR on the proliferation of colorectal cancer cells were mediated by inhibition of cell cycle progression, we followed cell cycle progression in SW480 and HCT116 cells with flow cytometry. After treatment with pCDNA-BANCR or empty vector for 48 h, the results demonstrated that BANCR over-expression led to a significant accumulation of cells at G0/G1-phase (p < 0.05) and a significant decrease in cells in S-phase compared with control (p < 0.05; Fig 3A and 3B).

Next, we investigated the effects of BANCR over-expression on apoptosis. As shown, the percentages of apoptotic cells were significantly increased in the treated group compared to control group (p < 0.05; Fig 3C and 3D). Taken together, BANCR treatment could induce colorectal cancer cell apoptosis and G0/G1 phase arrest.

### BANCR inhibits tumorigenesis of breast cancer cells in vivo

**Fig 4:**
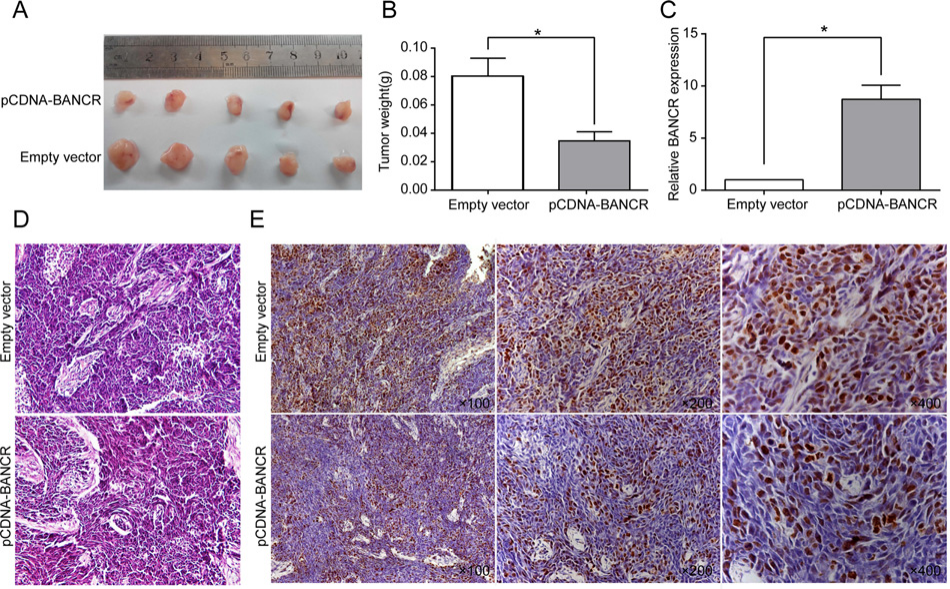
BANCR inhibits tumor growth in a xenograft mouse model. (A) Total number of tumors after removal from the mice. (B) Tumor weight when the tumors were harvested. The data represent the mean ± SD. *, P < 0.05. (C) qRT-PCR analyses indicated that BANCR expression was significantly increased in vivo. The data represent the mean ± SD. *, P < 0.05. (D, E) Representative images of HE staining and immunohistochemistry of the tumor. The IHC showed an upregulation of BANCR and a decreased expression of the proliferation index Ki67.

We next validated the effects of BANCR over-expression on the proliferation of colorectal cancer cells in vivo. HCT116 cells transfected with pCDNA-BANCR or empty vector were injected into nude mice. Fifteen days after injection, we found that the tumors formed in the pCDNA-BANCR group were dramatically smaller than those in the empty vector group (Fig 4A). In addition, the average tumor weight was markedly lower in the pCDNA-BANCR group compared with the control group at the end of the experiment (p < 0.05, Fig 4B). qRT-PCR analysis of BANCR expression was then performed in selected tumor tissues. The results showed that the levels of BANCR expression in tumor tissues formed from pCDNA-BANCR cells were higher than those of the tumors formed in the control group (p < 0.01, Fig 4C). HE staining was observed under the microscope, and compared with the negative control, we found that karyopyknosis and shape change existed in the tumor sample treated with pCDNA-BANCR (Fig 4D). The proliferation index Ki67 determined by immunohistochemical staining was significantly decreased in the BANCR-transfected tumors (Fig 4E). These results indicate that BANCR over-expression could inhibit tumor growth in vivo.

### BANCR suppresses the proliferation of colorectal cancer cells by targeting p21

**Fig 5:**
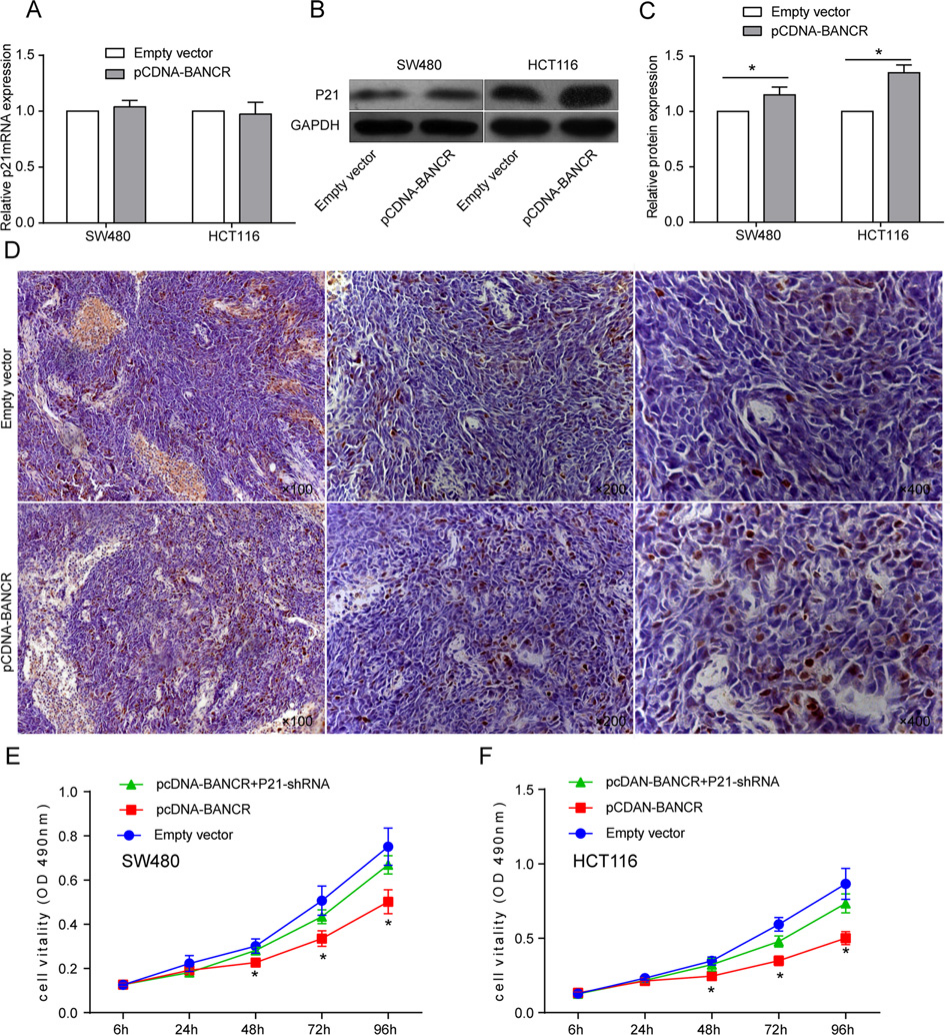
p21 is a key downstream mediator of BANCR. (A) The expression level of p21 mRNA was measured using qRT-PCR. (B, C) Analysis of p21 expression in sw480 and HCT116 cells treated with pCDNA-BANCR by Western blot. The results are from three independent experiments. GAPDH protein expression was used as an internal control. (D) The IHC showed an upregulation of BANCR increased p21 expression in a xenograft mouse model. (E F) The MTT assay was performed to determine the proliferation of SW480 and HCT116 cells transfected with empty vector, pcDNA-BANCR+p21-shRNA or pcDNA-BANCR. Data represent the mean ± S.D. from three independent experiments. *, P < 0.05.

Various mechanisms have been proposed to explain lncRNA-mediated gene expression, and one of the important ones is their ability to interact with p21 proteins[12,13]. To further explore whether p21 plays critical roles in BANCR mediated proliferation in colorectal cancer cells, qRT-PCR and Western blot assays were performed to detect the expression of p21 mRNA and p21 protein in SW480 and HCT116 cells after treatment with pCDNA-BANCR or empty vector. As shown in Fig 5A, The expression level of p21 mRNA did not change significantly (p ≥ 0.05), but p21 protein levels were significantly increased in the treated group compared with the control group (Fig 5B and 5C). Likewise, nude mice tumor xenograft immunostaining indicated that the positive rate of p21 protein in tumor tissues from pCDNA-BANCR transfected cells was significantly stronger than from empty vector transfected cells (Fig 5D).

To further verified whether p21 substantially contributed to the growth inhibition mediated by BANCR over-expression, we performed rescue experiments. After transfection with pCDNA-BANCR, SW480 and HCT116 cells were cotransfected with p21-shRNA. The effects of p21-shRNA and BANCR over-expression on cell proliferation were assessed using the MTT assay. We found that p21 knockdown partially compromised the effects of BANCR over-expression on colorectal cancer growth inhibition (Fig 5E and 5F). The results indicated that the effect of BANCR on colorectal cancer is at least in part through targeting P21.

## Discussion

Recently, genomic studies have revealed that < 2% of the total human genome can be transcribed, and these transcripts, including not only the well-known microRNAs (miRNAs) but also a heterogeneous group of so called long noncoding RNAs (lncRNAs) are emerging as critical regulators of gene expression and cell fate[14]. LncRNAs are non-coding RNAs greater than 200 nt in length and can be transcribed by RNA polymerase II (RNA pol II) and are polyadenylated[15,16]. Several association studies have recognized that they may function on various aspects of cell physiology and pathology, such as imprinting[17], maintenance of pluripotency[18], and cancer[19]. For example, lincRNA-p21, uc.73, and uc.338 have been reported to be correlated with human malignancies[19]. Indeed, the new study has found that lncRNAs are often deregulated in cancer cells compared with normal cells, and their dysregulation in cancer cells often induces apoptosis, suggesting that lncRNAs can be considered, at least for some cancer types, as therapeutic targets[20].

BRAF-activated non-coding RNA (BANCR) was first found via an RNA-seq screen for transcripts affected by the expression of the oncogene BRAFV600E[10,21]. Guo, Q et al. showed that BANCR was frequently overexpressed in colorectal cancer tissues and this overexpression was found to significantly correlate with lymph node metastasis and tumor stage[22]. However, in our study, we found that the expression of BANCR was significantly downregulated in colorectal cancer tissues, and it is likely to be due to individual difference. Additionally, we observed that 3 colorectal cancer cell lines exhibited low BANCR expression, which corroborate our colorectal cancer tissues findings.

As low BANCR expression was associated with tumor size in colorectal cancer, we speculated that BANCR could play a significant role in tumor cell proliferation. Indeed, our study showed that BANCR overexpression in SW480 and HCT116 cells inhibited cell proliferation and caused a dramatic decrease in colony formation. The result was also confirmed by Edu analysis, HE staining and mouse xenograft studies, whereas knockdown of BANCR significantly enhanced cell proliferation in HCT116 cell lines. We next determined whether BANCR expression influenced tumor-like characteristics, such as cell cycle progression and apoptosis. After treatment with pCDNA-BANCR or empty vector for 48h, we tested cell cycle progression and apoptosis in SW480 and HCT116 cells with flow cytometry. The result demonstrated that BANCR over-expression led to a significant G1 arrest and a related increase in apoptosis. These results revealed that BANCR might impact the proliferation of colorectal cancer by affecting cell cycle progression and apoptosis.

It is widely accepted that the transformation of colon epithelial cells results from either silencing of tumor suppressor genes or activating oncogenes[23]. Tumor suppressor genes can negatively regulate cell proliferation by inducing growth arrest[24]. p21 is known as a universal inhibitor, which plays a role through the p53 dependent or independent pathway, resulting in cell cycle arrest at the G1 checkpoint and inhibition of further cell proliferation[25,26]. For example, p21 has been reported to lead to the inhibition of lung cancer cell proliferation by inducing G0/G1 arrest[27]. In addition, p21 has been reported to function under certain conditions as a potent antiapoptotic factor, acting at different levels of the death cascade[28]. For instance, treatment of A549 cells with the histone deacetylase inhibitor trichostatin A triggered apoptosis, and this response was accompanied by caspase-3-dependent cleavage of p21[29].

To explore the molecular mechanism through which BANCR led to the inhibition of cell proliferation and promotion of apoptosis of colorectal cancer, we analyzed BANCR over-expression and p21 mRNA or protein in sw480 and HCT116 cells, and we found that the expression of p21 mRNA did not change significantly, but p21 protein levels were significantly increased in the treated group compared with the control group. A similar result was also detected in nude mice tumor xenografts by immunohistochemistry. To further confirm that P21 was involved in the BANCR-induced decrease in colorectal cancer cell proliferation, we performed rescue experiments. The results showed that cotransfection with P21-shRNA and pCDNA-BANCR partially rescued the proliferation inhibition induced by BANCR over-expression, indicating that downregulated BANCR increased cell proliferation and inhibited cell apoptosis partially through P21.

In summary, in this study, we have shown that BANCR is downregulated in colorectal cancer tissues. The effects of this lncRNA on cell proliferation suggest that it promotes tumorigenesis in colorectal cancer. Finally, we showed that BANCR regulated cell proliferation by targeting p21, and its effects were likely posttranscriptional. Although our findings suggest that BANCR plays a role in the oncogenic process, tumor growth and progression, further studies in larger samples with a better distribution of each stage are required.
